# Overcoming the Straw Man Effect in Oncology: Visualization and Ranking of Chemotherapy Regimens Using an Information Theoretic Approach

**DOI:** 10.1200/CCI.17.00079

**Published:** 2017-11-15

**Authors:** Jeremy L. Warner, Peter C. Yang, Gil Alterovitz

**Affiliations:** **Jeremy L. Warner**, Vanderbilt University, Nashville, TN; **Peter C. Yang**, Massachusetts General Hospital; and **Gil Alterovitz**, Harvard Medical School and Harvard-Massachusetts Institute of Technology Division of Health Science, Boston; and Massachusetts Institute of Technology, Cambridge, MA.

## Abstract

**Purpose:**

Despite the plethora of randomized controlled trial (RCT) data, most cancer treatment recommendations are formulated by experts. Alternatively, network meta-analysis (NMA) is one method of analyzing multiple indirect treatment comparisons. However, NMA does not account for mixed end points or temporality. Previously, we described a prototype information theoretical approach for the construction of ranked chemotherapy treatment regimen networks. Here, we propose modifications to overcome an apparent straw man effect, where the most studied regimens were the most negatively valued.

**Methods:**

RCTs from two scenarios—upfront treatment of chronic myelogenous leukemia and relapsed/refractory multiple myeloma—were assembled into ranked networks using an automated algorithm based on effect sizes, statistical significance, surrogacy of end points, and time since RCT publication. Vertex and edge color, transparency, and size were used to visually analyze the network. This analysis led to the additional incorporation of value propagation.

**Results:**

A total of 18 regimens with 42 connections (chronic myelogenous leukemia) and 28 regimens with 25 connections (relapsed/refractory multiple myeloma) were analyzed. An initial negative correlation between vertex value and size was ameliorated after value propagation, although not eliminated. Updated rankings were in close agreement with published guidelines and NMAs.

**Conclusion:**

Straw man effects can distort the comparative efficacy of newer regimens at the expense of older regimens, which are often cheaper or less toxic. Using an automated method, we ameliorated this effect and produced rankings consistent with common practice and published guidelines in two distinct cancer settings. These findings are likely to be generalizable and suggest a new means of ranking efficacy in cancer trials.

## INTRODUCTION

Health care data can be highly convoluted, given the significant dimensionality, nonlinearity, and temporality present in most clinical contexts. In oncology, knowledge has been painstakingly built over decades, primarily through carefully designed randomized controlled trials (RCTs). RCT data, which evolve longitudinally over years and usually involve many indirect comparisons, are known to be subject to many potential biases, ones that can be difficult to discern.^1^ As a likely result of this complexity, the conventional approach to the ranking and recommendation of cancer treatments studied in RCTs has been expert consensus–driven guidelines (eg, the National Comprehensive Cancer Network [NCCN] guidelines). Alternatively, work by others has shown that network meta-analysis applied to RCTs can yield powerful insights^2-7^; however, the networks in these studies have been relatively simple, do not allow for mixed end points (eg, overall survival and response rate), and do not account for temporal factors. In complex networks, layout, animation, and visual parameters such as size and color take on increasing importance.^8^ For example, visual analytics have been successfully applied to temporal associations of laboratory results, phenotype relationship networks, and patterns of publication by biomedical specialty and primary degree.^9-11^ Visual analysis of networked RCT data may help uncover previously underappreciated biases.

In previous work, we described a prototype approach for the automated construction of a ranked chemotherapy treatment regimen network using information-theoretical techniques, which were applied to the first-line treatment of chronic myelogenous leukemia (CML-1).^12^ Here, we demonstrate how extension of the approach through additional information theoretical measures help overcome the apparent presence of a straw man phenomenon. The straw man effect is a bias that causes new studies to appear more promising because they are compared with regimens that are comparatively ineffective.^1^ Although this bias has been described, the degree to which it pervades clinical trial design is unknown. The objective of this paper is to present a new algorithm, built on prior foundations,^12^ as well as to visually analyze this putative straw man phenomenon in the CML-1 scenario and a second scenario, the treatment of relapsed/refractory multiple myeloma (RRMM).

## METHODS

### Context-Specific Regimen Identification

The RCTs that were previously identified in the context of CML-1 were also used in this study, along with several newly published RCTs. Briefly, RCTs were identified through a PubMed query and by hand searches of the literature and published guidelines. There were 27 RCTs identified between 1968 and 2016, with 18 distinct regimens, representing 10,282 patients studied (Data Supplement). To identify RCTs for the context of RRMM, we used a combination of an established knowledge base of chemotherapy regimens, HemOnc.org,^13^ along with RCTs identified by two recent network meta-analyses in this setting.^6,7^ This yielded a total of 25 RCTs published between 2004 and 2016 containing outcome information for 28 distinct regimens, representing 9,737 patients studied (Data Supplement).

### Algorithm Modifications

The previous valuation algorithm,^12^ which was used for ranking as well as for coloration of vertices, was revised to include strength of evidence, effect sizes, aging effects, propagation, and refresh as explained in the following paragraphs.

#### Efficacy measure.

For all trials, we selected the trial-defined primary end point, as described in the published manuscript, as the main efficacy measure for the valuation algorithm. For trials with more than one predefined primary end point, we used the least-surrogate end point. If the primary end point was met, we used less-surrogate secondary end points in the algorithm if they had marginal or better statistical significance (ie, *P* ≤ .10).^14^ Conversely, if the primary end point was not met but secondary end points were met, we still used the primary end point for the valuation algorithm. We assigned a relative value (RV) as follows: 1.0 for strong, 0.8 for intermediate, and 0.7 for weak end points (Table 1; Equation 1). To determine the stability of the rankings, we varied RV by ±5%, ±10%, and ±20% in a sensitivity analysis.

**Table 1.**
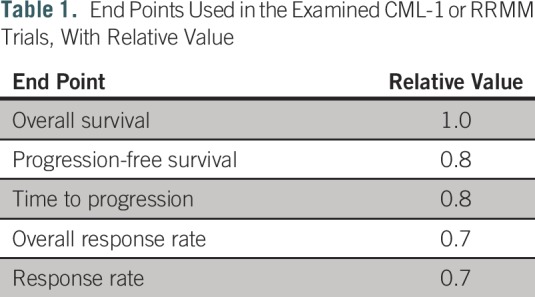
End Points Used in the Examined CML-1 or RRMM Trials, With Relative Value

#### Strength of evidence.

In our pilot work,^12^ we used a simple win-lose-draw framework with win and lose defined as a superior or inferior finding with a *P* value ≤ .05, and draw defined as statistical nonsignificance or formal noninferiority.^15^ Here, we introduce a weighted entropy measure: the negative logarithm of the *P* value. Because very small *P* values are difficult to interpret, this coefficient is allowed to take a maximum value of 3 (ie, *P* values <.001 were truncated to .001).

#### Effect size.

We replaced the win-lose-draw framework with a coefficient representing the effect size reported in the trial. For time-based outcomes (eg, overall survival), we ideally used the hazard ratio (HR) as the effect size.^16^ When HR was not reported, we defined the effect size either as the ratio of the median survival times or as the point estimate reported as significant in the publication (eg, the 3-year event-free survival). For nontemporal measures (eg, response rate), we used the calculated odds ratio as the effect size. In all cases, the effect size > 1 was transformed into a coefficient *E*, which is positive for the winning side and negative for the losing side (eg, if a publication reports HR = 0.5, *E* = 2 for the winning side and *E* = −2 for the losing side).

#### Aging effects.

To incorporate outdating of scientific evidence, we introduced an exponential decay coefficient as a function of the time since publication of trial results; additional details are in the Data Supplement.

#### Vertex valuation algorithm.

After incorporation of strength of evidence, effect size, and aging effects, the empirical vertex valuation formula is as follows:

Equation 1 $${\hat{v}}_{n}=\sum_{y=1}^{m}-\log_{10}\left(P_{y}\right)\times RV_{y}\times E_{y}\times \log_{10}\left(N_{y}\right)\times f\left(\alpha_{y}\right)$$

where for the *n*th vertex *v*, there are *m* incident edges, *E* is the effect size coefficient of the *y*th edge, *N* is the total number of patients in each pairwise comparison, *P* is the *P* value of the *y*th outcome, and $f\left(\alpha_{y}\right)$ is the aging coefficient described in the previous paragraph. A positively valued vertex is considered recommendable, and a negatively valued vertex is considered contraindicated. A vertex with value near zero is considered to have lacking evidence, conflicting results, or poor study quality such that there is insufficient evidence on whether to recommend. Although the valuation coefficient is unitless, the magnitude informs the power of the valuation and, thus, it is not normalized.

#### Propagation and refresh.

To overcome the apparent straw man effect (discussed in Results), we investigated the introduction of indirect evidence propagation. In our pilot work, we did not assign any node valuation on the basis of indirect evidence, such that the calculated network is akin to a single-layer perceptron (aka, pairwise network analysis). We augmented this model with information propagation, which has been studied in the context of social networks.^17-19^ Specifically, we allow nodes that were calculated to lose value as a result of newly introduced evidence to pass some of their value loss to regimens to which they had previously been superior (ie, single-generation value propagation). Conversely, nodes that were calculated to gain value as a result of newly introduced evidence pass some of their value gain to regimens to which they had previously been inferior. For example, in CML-1, dasatinib was demonstrated to be superior to imatinib^20^; however, imatinib had been shown 7 years earlier to be superior to interferon α and low-dose cytarabine (IFNA/LoDAC).^21^ Therefore, a portion of the value loss assigned to the imatinib node is propagated to the IFNA/LoDAC node. This has the effect of restoring some value to imatinib. When value is propagated under these constraints, we also refresh the age-related devaluing coefficient by one half-life. This has the simultaneous effect of allowing more value to be propagated while also restoring some relevance to the older regimen; this is analogous to decreasing impedance in an electrical circuit, and is one possible solution to the problem of hindsight bias.^22^ See the Data Supplement for a more detailed, graphical description.

### Treatment Network Visualization

We used multiple visual variables to display the treatment regimen networks: size, color, transparency, and position.^23^ See the Data Supplement for details.

### Statistical and General Methods

R version 3.4.0 and RStudio version 1.0.143 (https://www.r-project.org/) were used for the calculations. Graphs were created and displayed using the igraph package version 1.0.1^24^ (http://igraph.org/r/); coloration was by the RColorBrewer package.^25^ Correlations between vertex value and size before and after value propagation were calculated using the Pearson product-moment correlation; unadjusted *P* values < .05 were considered statistically significant. Animations of all networks and the R code used to develop them are available upon request.

## RESULTS

### Visualization of the Treatment Regimen Networks

**Fig 1.**
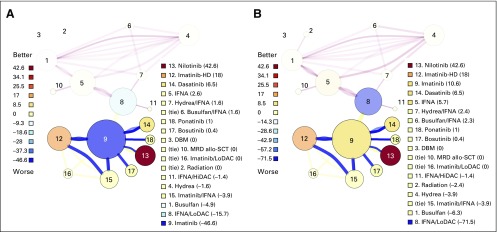
Chemotherapy regimen network for first-line treatment of chronic myelogenous leukemia-1 (CML-1), through 2016. (A) Initial valuations, before application of propagation and refresh. The current standard of care for CML is the use of tyrosine kinase inhibitors (TKIs) in the upfront setting. Consistent with this, TKIs are highly ranked, with the exception of imatinib, which is the lowest-ranked regimen. (B) Applying propagation and refresh to the network changes several valuations, most notably imatinib. DBM, dibromomannitol; HD, high dose; HiDAC, high-dose cytarabine; IFNA, interferon α; LoDAC, low-dose cytarabine; MRD allo-SCT, matched related-donor allogeneic stem-cell transplant.

**Fig 2.**
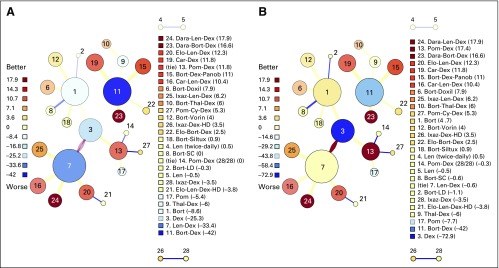
Chemotherapy regimen network for treatment of relapsed/refractory multiple myeloma, through 2016. (A) Initial valuations, before application of propagation and refresh. The current standard of care is doublet or triplet therapy using an immunomodulatory drug or proteasome inhibitor backbone. This is reflected in the ranking, although Bort-Dex and Len-Dex are the lowest-ranked regimens. (B) Applying propagation and refresh to the network changes several valuations; most notably, Dex is much more negatively valued. Bort, bortezomib; Car, carfilzomib; Cy, cyclophosphamide; Dara, daratumumab; Dex, dexamethasone; Elo, elotuzumab; HD, high dose; Ixaz, ixazomib; LD, low dose; Len, lenalidomide; Panob, panobinostat; Pom, pomalidomide; SC, subcutaneous; Siltux, siltuximab; Thal, thalidomide; Vorin, vorinostat.

The resultant networks for CML-1 and RRMM in the most recent year of analysis (2016), after incorporation of evidence strength, effect size, and aging into the valuation algorithm, are shown in Figures 1A and 2A; a complete list of regimens and the number of patients studied for each are shown in the Data Supplement. On initial visualization of the CML-1 regimen network, a few things are immediately evident: (1) there are severe aging effects on regimens 1 through 8, with most of these being valued somewhere near zero; (2) the quality of the outcome measure degrades over time, with the newer regimens almost exclusively evaluated with weak surrogate end points (blue edges); and (3) the largest vertex, regimen 9 (imatinib), is also the lowest ranked. Visualization of the RRMM network reveals that (1) aging effects only seem prominent for regimens 1 through 5; (2) outcome measures are mostly intermediate surrogates (eg, progression-free survival); and (3) the largest and most connected regimens are the lowest ranked. The visually apparent link among connectedness, size, and low valuation on the initial visual inspection led us to suspect that straw man effects were present in both networks, but potentially overstated.

### Uncovering and Countering the Straw Man Effect

**Fig 3.**
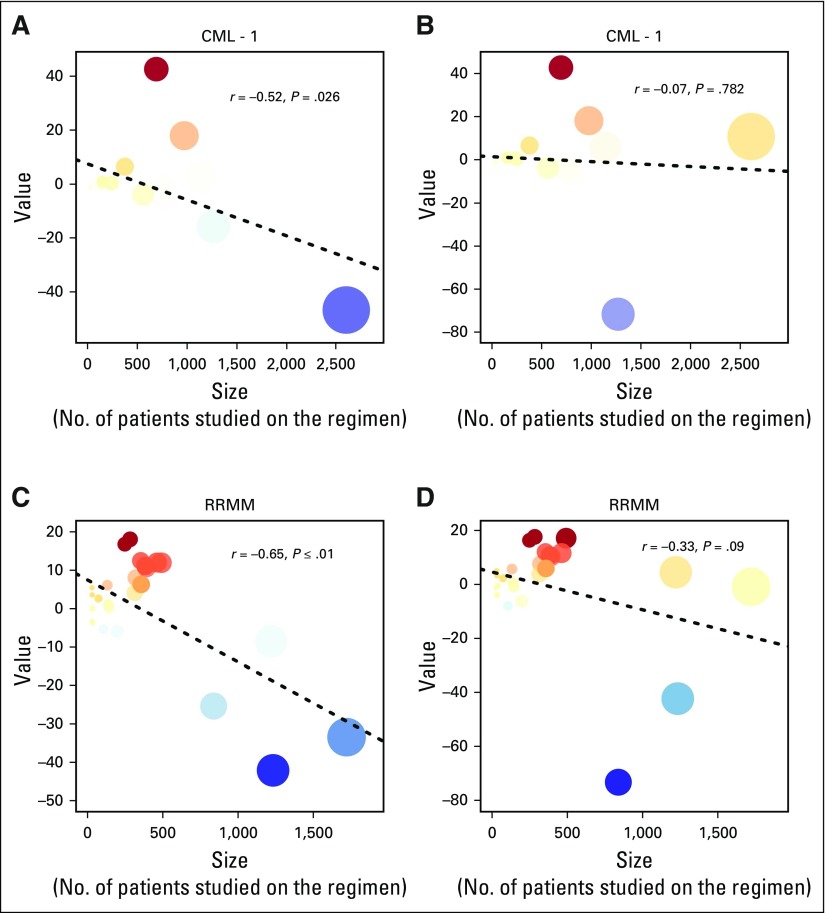
Scatterplots of vertex value versus size for CML-1 and RRMM. Linear regression lines using the least squares approach are overlaid. For all panels, the results show that value is inversely correlated with size, although the correlation becomes nonsignificant once propagation and refresh are instituted. Proportionate size and transparency are preserved from the treatment network visualization; numbering of regimens is omitted to improve clarity. CML-1, chronic myelogenous leukemia; RRMM, relapsed/refractory multiple myeloma.

When the vertices are plotted by vertex value versus size (ie, the total number of patients studied under the regimen), the apparent tendency for large vertices to be negatively valued becomes more evident, as shown in Figure 3. In both contexts, this negative correlation was initially statistically significant: For CML-1, the value of *r* for the correlation of value and size was −0.52 (95% CI, −0.80 to −0.07; *P* = .026). For RRMM, the value of *r* for the correlation of value and size was−0.65 (95% CI, −0.82 to −0.37; *P* = .0002).

Upon inclusion of propagation and refresh, the valuation of some vertices changes dramatically, as shown in Figures 1B and 2B. In the CML-1 network, imatinib moves from the lowest-ranked regimen to the regimen ranked third highest; IFNA/LoDAC inherits most of the negative value from imatinib and becomes the lowest-ranked regimen. In the RRMM network, almost all aging effects disappear due to refresh, bortezomib and lenalidomide -dexamethasone become more positively valued, dexamethasone (Dex) becomes even more negatively valued, and pomalidomide-dexamethasone (Pom-Dex) moves from the fourth-highest ranked regimen to the second-highest ranked. With this adjustment, the correlation between value and size changes and is no longer significant; for CML-1, the value of *r* for the correlation between value and size becomes −0.07 (95% CI, −0.52 to 0.41; *P* = .78). For RRMM, the value of *r* for the correlation of value and size becomes −0.33 (95% CI, −0.62 to 0.05; *P* = .09).

### Sensitivity Analysis

With systematic variation in RV, the magnitudes of the vertex values changed slightly, but the rank order did not change for CML-1 or RRMM. Positively valued regimens remained positively valued and vice versa. See the Data Supplement.

## DISCUSSION

The interpretation of complex networked data benefits from computational approaches and visualization of the results. In the examples discussed here, multiple visual channels (ie, color, transparency, size, position) provided an integrated picture of context-specific treatment scenarios that evolved over many years (CML-1, 49 years; RRMM, 13 years). We were able to leverage human color perception through the use of a divergent color scale,^26^ as compared with the rainbow color map often used in scientific visualizations.^27^ The human visual system is particularly well adapted for anomaly detection, owing to enhanced perception of color, edges, and outliers.^28,29^ As such, we were able to immediately recognize a potential anomaly in that the largest nodes in the CML-1 and RRMM networks seemed to be both highly connected (ie, compared with many other regimens) and negatively valued. This evidence from visual inspection led to further investigation into a possible straw man effect, which was initially supported by the existence of a statistically significant negative correlation between vertex value and size for both contexts. Through the introduction of propagation and refresh into our algorithm, we were able to ameliorate the straw man effect, although it was not eliminated entirely.

Generally, the straw man effect is most evident when new interventions are compared with clearly inferior regimens.^1,30^ A subtler version is the tendency to compare a new regimen with a comparatively effective regimen using a weaker surrogate end point, such as progression-free survival.^31-33^ It has been suggested that pharmaceutical industry support, along with reluctance to sponsor head-to-head comparisons of drugs manufactured by perceived competitors, may exacerbate such effects.^34,35^ Two examples where the straw man effect may be operational in cancer domains other than those examined here are dacarbazine in melanoma, where at least nine RCTs demonstrating inferiority have been published between 2000 and 2017^36^; and docetaxel in non–small-cell lung cancer, where at least eight RCTs demonstrating inferiority have been published between 2014 and 2017.^37^ The straw man effect is particularly hard to identify directly from the medical literature because the design and execution of RCTs may precede their publication by years. Also, many contexts have the fortunate situation in which prognosis is improving as a result of treatment, including both CML-1^38,39^ and RRMM^40^; this will naturally lead to the need to substitute surrogate end points so that new RCTs can be completed within a reasonable time. An intriguing possible way to mitigate the biases of RCT design is the use of a treatment of physician’s choice control arm, which was used in the recent (negative) CheckMate 026 trial.^41,42^

Any algorithmic ranking algorithm must be judged on face validity. In the final CML-1 network (Fig 1B), the algorithm ranks nilotinib, imatinib (standard and high dose), and dasatinib the highest, in close concordance with NCCN guidelines.^43^ Imatinib, in particular, is the subject of ongoing contention for many reasons, including (1) all regimens prospectively evaluated against imatinib subsequent to the IRIS (International Randomized Study of Interferon and STI571) trial^21^ have either been neutral or superior to imatinib; (2) surrogate end points have been substituted extensively; and (3) imatinib is now generic and cost may be lower than treatments still under patent protection.^44^ Given these challenges, it is notable that our updated algorithm still ranks imatinib highly. In the final RRMM network (Fig 2B), the algorithm ranks DRd as the highest and Dex as the lowest ranked regimen, in agreement with the analyses by Botta et al^6^ and van Beurden-Tan et al.^7^ Interestingly, our algorithm ranks a doublet, Pom-Dex, as second-highest, just behind Dara-Len-Dex. This is primarily due to the refresh of Dex, which allows for substantial negative value to be propagated from Pom-Dex to Dex. The validity of this finding is supported by the fact that Pom-Dex has a category 1 recommendation by the NCCN, although it is noted by the NCCN that triplet regimens are preferred to doublet regimens except in frail or elderly patients.^45^ Notably, the only triplet compared with Pom-Dex, pomalidomide-cyclophosphamide-dexamethasone, was in a randomized phase II trial with a weak surrogate primary end point (overall response rate) and borderline significance (*P* = .035).^46^ Given that Pom-Dex is relatively well tolerated,^47,48^ and that toxicities are a serious concern for modern myeloma drugs,^49^ our findings may have real-world significance.

There are several limitations to our current approach. First and foremost, significant methodological challenges remain in the field of dynamic network analysis, especially with respect to in-study and between-study effect modifiers.^50-52^ The vertex valuation algorithm contains several empirically derived coefficients and, therefore, could be subject to unmeasured bias. However, it is notable that the ASCO Value Framework has adopted similar weighting metrics to those we use for the surrogacy of end points.^53,54^ The ASCO Value Framework and other approaches to comparative valuation, such at the NCCN Evidence Blocks,^55^ are also empirically derived. We have used several definitions for effect sizes, including using point estimates when HRs are not reported. Older publications are more likely to report point estimates rather than HRs, so this could introduce a time-based systematic bias.^56^ In recognition of this limitation, we support efforts such as SAMPL to encourage uniform reporting of HRs.^57^ Current work is focused (as future work will be) on the use of ensemble methods to evaluate how rankings change with perturbation of the weighting coefficients, with a goal of choosing a consensus model that represents best fit.^58^ Beyond this methodological limitation, the measured valuations may also be subject to positive publication bias (ie, RCTs that demonstrate a statistically significant result are more likely to be published).^59^ We have tried to ameliorate the known tendency for positive publication bias by including so-called gray literature when possible. Future work will investigate incorporating information from clinical trial registries (eg, ClinicalTrials.gov) so that unpublished trials might be incorporated into the model. It is also possible that supplementation with comparative effectiveness data may change the valuations and subsequent rankings. We will also investigate more granular definitions of time of publication and incorporate evidence updates into the valuation algorithm, as opposed to the current method of using interim updates only to modify the aging coefficient.

In conclusion, we have described how the creation and visual inspection of chemotherapy treatment regimen networks can rapidly lead to new insights. In both of the described use cases, the visual variables of color, transparency, size, and position led to an almost immediate recognition of an anomaly that appeared to be the manifestation of the straw man effect. Additional modifications to the algorithm led to hierarchical rankings with face validity for both scenarios. These findings are likely to be generalizable to any cancer setting with multiple indirect comparisons, and they suggest a new means of ranking efficacy in cancer trials.
